# *ADIPOQ* single nucleotide polymorphisms and breast cancer in northeastern Mexican women

**DOI:** 10.1186/s12881-020-01125-8

**Published:** 2020-09-25

**Authors:** Ricardo M. Cerda-Flores, Karen Paola Camarillo-Cárdenas, Gabriela Gutiérrez-Orozco, Mónica Patricia Villarreal-Vela, Raquel Garza-Guajardo, Marco Antonio Ponce-Camacho, Ana Lilia Castruita-Ávila, Juan Francisco González-Guerrero, Iram Pablo Rodríguez-Sánchez, Ana Laura Calderón-Garcidueñas, Hazyadee Frecia Rodríguez-Gutierrez, Juan Carlos Arellano-Barrientos, Oscar Vidal Gutierrez, Hugo Alberto Barrera Saldaña, María Lourdes Garza-Rodríguez

**Affiliations:** 1 Facultad de Enfermería, Universidad Autonoma de Nuevo Leon, Monterrey, Nuevo León Mexico; 2 Facultad de Ciencias Biológicas, Universidad Autonoma de Nuevo Leon, Monterrey, Nuevo León Mexico; 3 Facultad de Medicina y Hospital Universitario “Dr. José Eleuterio González”, Departamento de Bioquímica Monterrey, Universidad Autonoma de Nuevo Leon, Monterrey, Nuevo León Mexico; 4 Facultad de Medicina y Hospital Universitario “Dr. José Eleuterio González”, Servicio de Anatomía Patológica y Citopatología, Universidad Autonoma de Nuevo Leon, Monterrey, Nuevo León Mexico; 5 Mexican Institute of Social Security, Hospital de Especialidades No 25, Monterrey, Nuevo León Mexico; 6 Facultad de Medicina y Hospital Universitario “Dr. José Eleuterio González”, Centro Universitario Contra el Cáncer (CUCC), Universidad Autonoma de Nuevo Leon, Monterrey, Nuevo León Mexico; 7 Facultad de Medicina, Universidad Veracruzana, Boca del Río, Veracruz, Mexico; 8 Vitagénesis SA, Monterrey, Nuevo León Mexico; 9 LANSEIDI FarbBiotec at Innbiogem, Monterrey, Nuevo León Mexico; 10 Centro de Biotecnología Genómica del Instituto Politécnico Nacional (IPN), Reynosa, Tamaulipas Mexico

**Keywords:** Breast cancer, Single nucleotide polymorphisms, Adiponectin, *ADIPOQ*, Mexican women

## Abstract

**Background:**

Adiponectin gene (*ADIPOQ*) polymorphisms have been shown to affect adiponectin serum concentration and some have been associated with breast cancer (BC) risk. The aims of this study were to describe the frequency of single nucleotide polymorphisms (SNPs) of *ADIPOQ* in Mexican women with BC and to determine if they show an association with it.

**Methods:**

DNA samples from 397 patients and 355 controls were tested for the *ADIPOQ* gene SNPs: rs2241766 (GT) and rs1501299 (GT) by TaqMan allelic discrimination assay. Hardy–Weinberg equilibrium (HWE) was tested. Multiple SNP inheritance models adjusted by age and body mass index (BMI) were examined for the SNP rs1501299.

**Results:**

We found that in the frequency analysis of rs1501299 without adjusting the BMI and age, the genotype distribution had a statistically significant difference (*P* = 0.003). The T allele was associated with a BC risk (OR, 1.99; 95% CI 1.13–3.51, TT vs. GG; OR, 1.53; 95% CI 1.12–2.09, GT vs. GG). The SNP rs2241766 was in HW disequilibrium in controls. In conclusion, the rs1501299 polymorphism is associated with a BC risk.

**Conclusions:**

Identification of the genotype of these polymorphisms in patients with BC can contribute to integrate the risk profile in both patients and their relatives as part of a comprehensive approach and increasingly more personalized medicine.

## Background

Breast cancer (BC) is the most common cancer in adult women in the world [1]. Different risk factors have been implicated in BC initiation and progression such as overweight (OW) and obesity (OB) [2]. OB is increasingly recognized as an oncogenic factor and is associated with many metabolic disorders, such as type 2 diabetes mellitus, coronary heart disease, and hypertension, and with cancer in different tissues, such as colon, prostate, and breast [3–5]. Moreover, it has been shown that excess adipose tissue promotes metastasis and recurrence of BC and is associated with increased mortality.

Adipocytes produce adiponectin and leptin, two highly expressed adipokines that have opposing effects on immune cell function [6]. Adiponectin is secreted exclusively by adipose tissue and has antineoplastic, anti-inflammatory, anti-oxidant, and anti-apoptotic roles [7]. This hormone also regulates estrogen, tumor necrosis factor (TNF), and insulin growth factor (IGF) secretion [6, 8–10]. Adiponectin serum concentrations are decreased in obese and diabetic subjects [10]. Studies support evidence that decreased adiponectin serum concentration may explain the increased risk of BC in OB^9^ and that this hormone is a potential diagnostic and prognostic BC biomarker [9].

*ADIPOQ* polymorphisms have been shown to affect adiponectin serum concentrations, and some have been associated with BC risk [10–12]. Adipokines are associated with several types of obesity-related cancers [13–17]. Previous studies found that homozygous carriers of the T allele of SNP rs1501299 had the highest concentration of adiponectin compared to the GG or TG genotypes [10, 18]. Also, an association of the rs2241766 TG and GG genotypes with increased adiponectin serum concentration and with a decreased risk for breast cancer was reported [10]. SNPs that cause lower adiponectin serum concentrations are associated with increased cancer risk; also, adiponectin levels are inversely correlated with adiposity. Decreased adiponectin serum concentrations may explain the increased risk of breast cancer in obesity [10, 18, 19].

The objectives of this hospital case-control study were: 1. To describe the frequency of two SNPs, rs2241766 (+ 45 T > G) and rs1501299 (+ 276G > T) of *ADIPOQ* in a sample of Mexican women from northeastern Mexico with and without BC, and 2. To determine any association between these polymorphisms and BC risk.

## Methods

### Approval from the scientific and ethics committees

The study was conducted in accordance with the Declaration of Helsinki, and the protocol was approved by the Institutional Ethics Committee of the University Hospital (registration no. BI10–002). All patients were invited to participate in the research project, an interview was performed and once the patients agreed to participate, they signed an informed consent. Afterwards, clinical and epidemiological information was collected, and blood samples were taken.

### Study design and population

A hospital case-control study was carried out. Patients were selected among women receiving chemotherapy at two institutions located in Monterrey City, Mexico: the Centro Universitario Contra el Cáncer (Hospital Universitario “Dr. José E. González” (HU), of the Universidad Autónoma de Nuevo León) and the Hospital de Especialidades #25 (Instituto Mexicano del Seguro Social (IMSS)). Both are referral centers for patients affected by this neoplasm who come from five states located in Northeast Mexico (Nuevo León, San Luis Potosi, Zacatecas, Coahuila, and Tamaulipas).

### Patient group

All BC patients were older than 18 years, had a confirmatory biopsy, and accepted to participate in the study and signed an informed consent. Because we are studying a low-penetrance gene (*ADIPOQ*), the study included only patients with sporadic breast cancer, thus, patients with a family history of BC were excluded. Pregnant women were also excluded.

Female BC patients attending the oncology clinics of the two participating hospitals who fulfilled inclusion criteria were invited by the oncologist to participate in the project. An interview was conducted to explain the protocol to the patients and subsequently, women who accepted to participate signed the consent letter. Blood samples were collected in the areas of chemotherapy and consultation. Epidemiological and clinical information was also collected. Only three patients refused to participate in the study. Most of the patients of the case group 288 (72.5%) and all the controls 355 (100%) came from the IMSS, the rest of the cases came from the HU (109/ 27.5%).

### Control group

Controls were women older than 18 years of age, without a history of cancer and a BI-RADS 1–2 mammogram who signed an informed consent. Individuals with a family history of BC and pregnant women were excluded.

All control group women were recruited in the radiology areas. They attended a follow-up mammography or were referred for timely detection of cancer by mammography. If the women fulfilled the inclusion criteria, they were invited to participate by interview and after signing the informed consent, a blood sample was taken in the radiology area and epidemiological and clinical information was collected. All 397 patients and 355 controls were Mexican women whose four grandparents were born in Northeast Mexico (Nuevo León, San Luis Potosi, Zacatecas, Coahuila, and Tamaulipas).

### DNA preparation

Genomic DNA was extracted from whole peripheral blood either with the QIAmp DNA Blood Kit (Cat No. 51104 Qiagen Inc., CA, USA) according to the manufacturer’s instructions, or using the TSNT method followed by phenol–chloroform extraction and ethanol precipitation [20]. DNA concentrations was measured by NanoDrop 8000 Spectrophotometer (Thermo Scientific, USA). Concentrations was adjusted to 50 ng/μl in purified nuclease-free water.

### Analysis of DNA polymorphisms

Samples from patients (*n* = 397) and controls (*n* = 355) were tested for the *ADIPOQ* gene SNPs: rs2241766 (GT) and rs1501299 (GT). All assayed polymorphic sites were genotyped using the TaqMan allelic discrimination assay (Assay ID: C__26426077_10 for rs2241766 and C___7497299_10 for rs1501299, Applied Biosystems, USA). All polymerase chain reactions (PCRs) were done in a volume of 10 μl containing: 5 μl of TaqMan universal PCR Master Mix 2X, 2 μl of DNA (100 ng), 0.2 μl of TaqMan SNP Genotyping Assays 20X, and 2.8 μl of nuclease free water. Thermal cycling conditions were 10 min at 95 °C, and 42 cycles each of 95 °C for 15 s, and 60 °C for 1 min. The Step One Real Time System (Applied Biosystems, USA) was used for genotyping.

### Statistical analysis

IBM SPSS version 24.0 was used for descriptive statistical analysis. First, participant characteristics were summarized as arithmetic means with standard deviation (SD) for continuous variables and counts and percentages for categorical variables. Second, the *p*-values of continuous variables were determined by unpaired *t-test*, for categorical variables, a chi- square test was used. Third, Hardy–Weinberg equilibrium (HWE) was tested with the public software developed by Tim M Strom and Thomas F. Wienker (http://ihg.gsf.de/cgi-bin/hw/hwa1.pl) without adjustment by age and BMI. Fourth, Multiple SNP inheritance models (codominant, dominant, recessive, overdominant and Log-additive) adjusted by age and BMI were examined to determine odds ratio (OR) and 95% confidence intervals (CI) (http://bioinfo.iconcologia.net/snpstats/start.htm). Given that this software has a limited sample size to run, a random woman was selected in cases (*n* = 258 of 397) and control (*n* = 258 of 355) groups; in order to randomly select, the EPIDAT package V4.1 was used. A *p-value* less than 0.05 was considered significant.

## Results

### Clinical and pathological characteristics

Demographic and clinical characteristics of the study population are shown in Table 1. The mean ages (±SD) were 52.4 (±12.0) and 49.5 (±10.9) for cases (*n* = 397) and controls (*n* = 355), respectively. Of the 12 characteristics analyzed, age at baseline interview, BMI, breast-feeding duration, menopausal status, and use of oral contraceptives showed statistical differences. Pathological characteristics are shown in the supplementary material.

**Table 1:** Comparison of 12 characteristics in case and control groups

Characteristics	(***n*** = 752)		***p***-value
	Case (***n*** = 397)	Control (***n*** = 355)	
	Mean ± SD or %	Mean ± SD or %	
Demographic factors			
**Age at baseline interview (years)**	52.5 ± 12	49.5 ± 10.9	0.009^a^
**Height (m)**	1.56 ± 0.07	1.60 ± 0.07	0.871^a^
**BMI > 30 (kg/m**^**2**^**)**			0.001^b^
**Yes**	43.1	29.9	
**No**	50.1	68.7	
**n/a**	6.8	1.4	
Reproductive factors			
**Age at menarche (years)**	12.8 ± 1.6	12.7 ± 1.5	0.379^a^
**Age at first** childbirth **(years)**	23.1 ± 5.6	23.4 ± 5.3	0.398^a^
**Number of** children born alive	3.3 ± 2	3.2 ± 2.2	0.115^a^
**Children**			
**Yes**	85.4	88.7	0.509 ^b^
**No**	14.6	11.3	
**Breast-feeding**			0.052^b^
**≤ 30 years old**	56.4	47.0	
**≥ 30 years old**	7.8	4.8	
**n/a**	35.8	48.2	
**Breast-feeding duration (months)**	12.2 ± 13.0	14.2 ± 22.2	0.002^a^
**Menopausal status**			0.001^b^
**Yes**	63.2	44.2	
**No**	33.0	55.5	
**n/a**	3.8	0.3	
**Age at menopause**	44.9 ± 5.7	45.9 ± 6.3	0.797^a^
Hormonal factors			
**Use of oral contraceptive**			0.001^b^
**Yes**	30.2	21.4	
**No**	66.0	78.6	
**n/a**	3.8	0.0	

*SD* standard deviation; *BMI* Body mass index; *n/a* not available; t-test^a^, chi-square test^b^

Invasive ductal carcinoma (IDC) was the most frequent cancer type (71.8%). The most frequent clinical stage was IIIB with 17.1%. The most common site of metastasis was bone. The majority of cases had modified radical mastectomy (MRM) surgery (65.8%) and 2.5% of women had bilateral cancer at diagnosis. Supplementary Table 1.

For biopsies with available immunohistochemistry (*n* = 274) for estrogen and progesterone receptors and HER2-neu, the information is shown in Table 2. In 82 cases (30%), a triple negative profile was observed.

**Table 2:** Breast cancer tumor immunohistochemistry (*n* = 274)

Immunohistochemistry	ER status n (%)	PR status n (%)	Her-2 status n (%)	TNBC status n (%)
Positive	149 (54.4)	127 (46.4)	114 (41.6)	82 (29.9)
Negative	125 (45.6)	147 (53.6)	165 (57.0)	192 (70.1)

*ER* Estrogen receptor; *PR* Progesterone receptor, *TNBC* Triple Negative Breast Cancer

### The allele and genotype frequencies of ADIPOQ polymorphisms

The allele and genotype frequencies for the rs1501299 and rs2241766 polymorphisms are summarized in Table 3. To carry out this analysis, 258 cases and 258 controls were randomly select, without adjusted by age and BMI. No significant deviations from HWE were found for the SNP rs1501299. The SNP rs2241766 was in HW disequilibrium in control women (*p* = 0.005). When we performed the frequency analysis without adjusting the BMI and age, the genotype distribution exhibited a statistically significant difference between cases and controls (*P* = 0.003 and *P* = 0.0003 for rs1501299 and rs2241766, respectively) and the T allele of rs1501299 was associated with BC risk (OR 1.99; 95% CI 1.13–3.51, TT vs GG; OR 1.53; 95% CI 1.12–2.09, GT vs GG).

**Table 3:** Frequencies of adiponectin polymorphism in Mexican Women wihout adjusted by age and BMI

Polymorphism	Frequencies	Cases %	Controls %	*p*-value (cases vs control)	OR	95% IC
rs1501299 (+276 G > T)	Genotype	*n* = 397	*n* = 355			
	GG^S^	44.4	58.3	0.003	Reference	
	GT	45.6	35.5		1.53	1.12–2.09
	TT	10.0	6.2		1.99	1.12–3.51
	*X*^*2*^	0.434	0.231			
	p-value HWE	0.51	0.63			
	Allele					
	G	0.671	0.761			
	T	0.329	0.239			
rs2241766 (Gly15Gly, + 45 T > G)	Genotype	*n* = 397	*n* = 355			
	TT^S^	71.0	62.8	0.0003	Reference	
	TG	27.2	29.6		0.81	0.51–1.12
	GG	1.8	7.6		0.20	0.07–0.51
	*X*^*2*^	0.8386	7.887			
	p-value HWE	0.360	0.005			
	Allele					
	T	0.847	0.776			
	G	0.153	0.224			

*HWE* Hardy-Weinberg Equilibrium

Subsequently, we performed the multiple SNP inheritance models of rs1501299 adjusted by age and BMI. With the codominant model, the genotype distribution exhibited a statistically significant difference between cases and controls (*p* = 8e-04) and the T allele of rs1501299 was associated with BC protection (OR 0.55; 95% CI 0.34–0.80 GT and OR 0.39; 95% CI 0.20–0.76 TT) (Table 4).

**Table 4:** *ADIPOQ* rs1501299 polymorphism using the multiple SNP inheritance models adjusted by age and BMI

Model	Genotype	Cases % *n* = 258	Controls *n* = 258	OR (95% CI)	*p*- value	AIC
Codominant	GG	41.1	58.1	1.00	8e-04	684
	GT	47.3	35.7	0.55 (0.34–0.80)		
	TT	11.6	6.2	0.39 (0.20–0.76)		
Dominant	GG	41.1	58.1	1.00	3e-04	683.1
	GT - TT	58.9	41.9	0.52 (0.36–0.74)		
Reccesive	GG – GT	88.4	93.8	1.00	0.04	692.1
	TT	11.6	6.2	0.51 (0.27–0.98)		
Overdominant	GG – TT	52.7	64.3	1.00	0.012	690
	GT	47.3	35.7	0.63 (0.44–0.90)		
Log-additive	–	–	–	0.59 (0.45–0.78)	2e-04	682.5

The rs2241766 G allele (GT and GG genotypes) was associated with a decreased risk for BC (OR 0.20; 95% CI 0.07–0.51, GG vs TT; add the OR and 95% CI for TG). However, because rs2241766 was out of HWE, these results should be considered with caution. Because this polymorphism is not in HW equilibrium, no multiple SNP inheritance models analysis was made.

We compared our genotypic frequencies with the frequencies reported for a population of 46 Mexican individuals from the National Center for Biotechnology Information (NCBI) web page [21]. The SNP rs2241766 frequencies in the controls (0.22 G allele and 0.78 T allele) were compared with the reported frequencies for the NCBI Hispanic population (0.28 for allele G and 0.71 for allele T). A chi-square test was applied and a significant difference between both populations was found for rs2241766 (*X*^*2*^ = 10.70, *p* = 0.005 when comparing the cases and *X*^*2*^ = 106.28, *p* = 1 × 10–7 when comparing the controls). Also, rs1501299 frequencies in the controls (0.76 G allele and 0.24 T allele) were compared with the reported frequencies for the NCBI Hispanic population (0.76 for allele G and 0.24 for allele T). A chi-square test was applied, and we did not find a significant difference between both populations (*X*^*2*^ = 0.006, *p* = 0.997, *X*^*2*^ = 3.16, *p* = 0.206) respectively.

### Association of BMI with ADIPOQ genotypes

We found a statistically significant BMI difference between women with breast cancer and the control group (Table 1). In order to identify if the BMI was associated with the rs1501299 and rs2241766 genotypes, we performed a univariate general lineal model. The result between cases and controls was a F vale of 6.29 (*p* = 0.012), for the rs2241766 (F = 1.473, *p* = 0.338) and the s1501299 (F = 2.417, *p* = 0.131). When we realized the interactions between BMI and rs1501299 (f = 0.30, *p* = 0.77) and rs2241766 (f = 0.18, *p* = 0.84), no significance association was found.

## Discussion

In this study, we found an association between rs1501299 in *ADIPOQ* and BC risk in Mexican women with an OR of 1.53 (95% CI, 1.12–2.09) for the GT genotype and 1.99 (95% CI, 1.13–3.51) for TT vs. GG. On the other hand, when information was analyzed adjusted by BMI and age using the five models, all of them showed an OR lower than one, considering a protective association. Besides the codominant model showed the lowest significant *p*-value (8e-04) and an AIC value of 684.27 with an OR of 0.55 (95% CI 0.34–0.80) for GT genotype, and an OR of 0.39 (95% CI 0.20–0.76) for TT vs GG.

In 2013, Kaklamani et al. found that rs1501299 was associated with BC risk not only in Caucasian population, but also in African American women [11]. Kaklamani associated the genotypes GG and TG with risk of breast cancer and reported that the TT genotype increases circulating adiponectin serum concentrations; this could explain the protective effect of genotype TT observed in our study [11]. Another study reported that variation in the ADIPOQ gene has effects on other types of cancer. They found that the T variation may have a protective effect in the development of endometrial cancer [22]. There is another study with opposite results that report that the GG genotype was associated with a higher adiponectin serum concentration in Kuwait [18]. Gui et al. found no association between genotypes of rs1501299 and adiponectin [23]. Also, there are reports that show an inverse association between adiponectin concentrations within breast tumors and tumor stage [24]. To date, reports of the association of rs1501299 genotypes with serum adiponectin levels are contradictory. Al Khaldi et al. reported that the T allele and TT genotype of rs1501266 reduce adiponectin levels in serum [18].

Another study reports no association between *ADIPOQ* SNPs and BC in American white women [25]. Genotypes TG and GG of SNP rs2241766 have also been associated with a decreased risk for BC when compared to the TT genotype, the rs2241766 variation with the G allele and the TG and TG + GG genotypes may have a protective effect in ductal infiltrating breast cancer in Mexican women [26].

In our study, the rs2241766 polymorphism was in HW disequilibrium in controls, so it is not possible to confidently associate it with breast cancer risk. The allelic discrimination plots obtained in the real time PCR were clean and we did not find differences between duplicates.

Some authors have also reported HW disequilibrium in different populations for the rs2241766 polymorphism [24]. The main causes of HW disequilibrium in controls were selection bias or a competing risk of death associated with the mutant gene [25].

We performed a general lineal model to identify if the BMI was associated with the polymorphism. We were able to identify that the polymorphism is associated with breast cancer risk, but not the BMI.

In our study, the controls did not come from the general population but from the radiology area, which may have contributed to selection bias and may partly explain the genotypic imbalance in the case of rs2241766. Another disadvantage of this study is that we were unable to analyze serum ADIPOQ levels due to variability in pre-analytical processes.

Further studies are needed to examine the association of adiponectin serum concentrations with the different gene polymorphisms [10, 19], particularly in Mexican populations.

We estimated associations between SNPs in *ADIPOQ* and BC risk in a hospital case-control study of Mexican women. To our knowledge, our study is the largest in Mexican population (397 cases and 355 controls) evaluating *ADIPOQ* SNPs in BC risk.

The Mexican population is Mestizo, and it is important to characterize the distribution of BC risk polymorphisms. Detection of SNP rs1501299 in the Mexican population may play an important role as a BC risk biomarker. Other reports in Mexican women found a positive relation with SNP rs1501299, and the response to chemotherapeutic treatment in patients with BC [19].

Mexico is a developing country where the use of genetic tests is not routine and SNP detection will be useful to support potential benefits of personalized medicine for Mexican population.

## Conclusions

In conclusion, we confirmed that rs1501299 polymorphism is associated with BC risk in a large series of Mexican women. The identification of the genotype of these polymorphisms in patients with breast cancer can contribute to integrate the risk profile in both patients and their relatives as part of a comprehensive approach and an increasingly more personalized medicine.

## Supplementary information

**Additional file 1 Table S1**. Clinical-pathological characteristics in the case group.

## Data Availability

All data generated or analyzed during this study are included in this manuscript. The sequences of the SNP were obtained from the NCBI GenBank database (https://www.ncbi.nlm.nih.gov/genbank/). The reference genome was GRCh38.p12 and the accession numbers for SNP are: NG_021140.1:g.15430 T > G for rs2241766, and NG_021140.1:g.15661G > T for rs1501299.

## Abbreviations

ADIPOQ: Adiponectin gene
BC: Breast cancer
SNPs: Single nucleotide polymorphisms
HWE: Hardy-Weinberg equilibrium
BMI: Body mass index
OW: Overweight
OB: Obesity
TNF: Tumor necrosis factor
IGF: Insulin growth factor
IMSS: Instituto Mexicano del Seguro Social
SDs: Standard deviations
ORs: Odds ratios
CI: Confidence intervals
IDC: Invasive ductal carcinoma
MRM: Modified radical mastectomy
NCBI: National Center of Biotechnology Information
