# Supplementary material for: ADIPOQ single nucleotide polymorphisms and breast cancer in northeastern Mexican women
Source: BMC Med Genet. 2020 Sep 25;21:187. doi: 10.1186/s12881-020-01125-8 (PMC7519484; doi:10.1186/s12881-020-01125-8)
Supplement: Supplementary file 1 — Additional file 1 Table S1. Clinical-pathological characteristics in the case group. [file 12881_2020_1125_MOESM1_ESM.docx]

Supplementary Table 1. Clinical-pathological characteristics in the case group.

| Characteristics | **Cases %**  ***n*=397** |
| --- | --- |
| Breast cancer | |
| **Histological type**  **IDC**  **ILC**  **Other** | 71.8  6.3  21.9 |
| **Localization**  **Right breast**  **Left breast**  **Bilateral**  **n/a** | 44.6  49.4  2.5  3.5 |
| **Contralateral cancer**  **Yes**  **No**  **n/a** | 10.1  79.3  10.6 |
| **Metastasis**  **Yes**  **No**  **n/a** | 23.4  63.2  13.4 |
| **Metastasis to**  **Bone**  **Lung**  **Liver**  **Nervous system**  **Other** | 9.3  5.5  2.8  3.0  2.8 |
| **Cancer staging**  **I**  **IIA**  **IIIA**  **IIB**  **IIIB**  **IIIC**  **IV**  **n/a** | 3.8  12.8  16.4  15.9  17.1  3.5  8.0  22.4 |

n/a not available

SD, standard deviation; IDC, Invasive ductal carcinoma; ILC, Invasive lobular carcinoma; MRM, Modified Radical Mastectomy; n/a, not available.
